# Methotrexate and anti-tumor necrosis factor treatment improves endothelial function in patients with inflammatory arthritis

**DOI:** 10.1186/s13075-017-1439-1

**Published:** 2017-10-17

**Authors:** Gia Deyab, Ingrid Hokstad, Jon Elling Whist, Milada Cvancarova Smastuen, Stefan Agewall, Torstein Lyberg, Nicoletta Ronda, Knut Mikkelsen, Gunnbjorg Hjeltnes, Ivana Hollan

**Affiliations:** 1 Department of Medical Biochemistry, Innlandet Hospital Trust, Hamar, Norway; 2 Lillehammer Hospital for Rheumatic Diseases, Lillehammer, Norway; 3 Institution of Health Care, Health Science PhD Program, Oslo and Akershus University College, Oslo, Norway; 4 Oslo University Hospital, Ullevål, Oslo Norway; 5 Institute of Clinical Sciences, University of Oslo, Oslo, Norway; 6 Department of Medical Biochemistry, Oslo University Hospital, Ullevål, Oslo Norway; 7 Department of Food and Drug, University of Parma, Parma, Italy; 8 Department of Medicine, Innlandet Hospital Trust, Lillehammer, Norway; 9 Department of Medicine, Brigham and Women’s Hospital, Boston, USA; 10 Harvard Medical School, Boston, USA; 11 Department of Research, Innlandet Hospital Trust, Brumunddal, Norway

**Keywords:** Inflammatory arthritis, Methotrexate, Anti-tumor necrosis factor, Rheumatic arthritis, Spondyloarthritis

## Abstract

**Background:**

Inflammatory arthritis (IA), including rheumatoid arthritis (RA), ankylosing spondylitis (AS) and psoriatic arthritis (PsA), leads to increased cardiovascular disease occurrence probably due to atherosclerosis. One of the first stages in atherogenesis is endothelial dysfunction (ED). Therefore, we aimed to compare endothelial function (EF) in patients with IA, and to examine the effects of methotrexate (MTX) monotherapy and antitumor necrosis factor (anti-TNF) treatment with or without MTX comedication (anti-TNF ± MTX) on EF.

**Methods:**

From the PSARA observational study, all patients with RA (*n* = 64), PsA (*n* = 29), and AS (*n* = 20) were evaluated for EF. In patients with ED at baseline (*n* = 40), we evaluated changes in the Reactive Hyperemic Index (RHI) after 6 weeks and 6 months of antirheumatic therapy.

**Results:**

In IA patients with ED, RHI significantly improved after 6 weeks (*p* < 0.001) and 6 months (*p* < 0.001) of treatment, independent of changes in disease activity parameters. After 6 months, RHI had improved more in the MTX group than in the anti-TNF ± MTX group, and the difference remained statistically significant after adjustments for potential confounders. Among patients with active RA, AS, and PsA, those with AS appeared to have the worst endothelial function, although they were the youngest.

**Conclusion:**

Treatment with MTX and anti-TNF ± MTX was associated with a relatively fast improvement of EF in IA patients with ED, independent of change in disease activity. Therefore, modes of action other than the anti-inflammatory effect may contribute to the EF improvement. After 6 months, the EF improvement was more pronounced in the MTX group than in the anti-TNF ± MTX group.

**Trial registration:**

Clinicaltrials, NCT00902005. Registered on 13 May 2009.

## Background

Inflammatory arthritis (IA), including rheumatoid arthritis (RA), ankylosing spondylitis (AS), and psoriatic arthritis (PsA), has increased cardiovascular (CV) morbidity and mortality, probably due to cardiovascular disease (CVD) caused by atherosclerosis [1–5]. The first step in the development of atherosclerosis is endothelial dysfunction (ED) which is initially a reversible process [6]. Thus, improving endothelial function (EF) might be of great importance in preventing atherosclerosis. The endothelium has several vital homeostatic functions, including regulation of vascular tone and growth, thrombogenesis and thrombolysis, and interactions between platelets and leukocytes and the vessel wall. The endothelium secretes vasorelaxing (e.g., nitric oxide) and vasoconstricting (e.g., endothelin-1) substances in response to mechanical stress [6, 7]. ED is characterized by impaired ability of the artery to dilate in response to physical and chemical stimuli [8, 9]. Assessment of EF has been used to estimate the CV risk in IA patients [10, 11].

Clinical studies indicate that antirheumatic treatment, including methotrexate (MTX) and antitumor necrosis factor (anti-TNF) treatment, not only ameliorates disease activity but also reduces CV morbidity and mortality in RA patients [12, 13]. There is also evidence that anti-TNF treatment improves EF in RA, and reduces arterial stiffness and intima-media thickness in patients with RA, PsA, and AS [14, 15].

However, information on the effect of antirheumatic drugs on EF in AS and PsA patients is still limited. Therefore, the aim of this study was to compare EF in RA, AS, and PsA patients, and to examine the effect of antirheumatic treatment (MTX and/or anti-TNF) on EF in these patient groups.

## Methods

### Patients

We examined patients from the PSoriatic arthritis, Ankylosing spondylitis, Rheumatoid Arthritis (PSARA) study who completed 6 months of follow-up and in whom EF was measured (*n* = 113). Of the 114 patients who completed the study, one PsA patient was excluded because she was not able to adhere to the requirements of the EF measurement (smoked and did not sit still).

All patients in PSARA, an observational study, had been included at the Lillehammer Hospital for Rheumatic Diseases as described elsewhere [16]. Briefly, the inclusion criteria were: males and females with an age range 18–80 years; and PsA according to the Moll and Wright 1973 criteria [17], AS according to the modified New York diagnostic criteria for AS [18], or RA according to the ACR 1987 criteria [19], and clinical indication for starting with either MTX monotherapy or anti-TNF treatment with or without MTX comedication (anti-TNF ± MTX).

Exclusion criteria included lack of cooperability, any contraindication for MTX and anti-TNF, any significant infection (including subclinical tuberculosis), pregnancy or breastfeeding, congestive heart failure, use of systemic glucocorticoids > 10 mg/day during the last 2 weeks or anti-TNF during the last 4 weeks before the inclusion, and any chronic inflammatory disease other than RA, AS, or PsA.

All patients were Caucasian and were examined at baseline and after 6 weeks and 6 months of treatment.

### Treatment

Patients were either treated with MTX monotherapy or with anti-TNF ± MTX. The type and doses of antirheumatic treatment were decided by rheumatologists not involved in the study upon clinical judgment and in accordance with Norwegian guidelines. Doses were as follows: etanercept 50 mg subcutaneous injection once a week; infliximab 3–5 mg/kg intravenous injection at baseline, then following standard dosing regimen; adalimumab 40 mg subcutaneous injection every other week; MTX 15–25 mg orally once a week.

Norwegian guidelines consider MTX as a first-line antirheumatic treatment in patients with chronic peripheral arthritis, in particular RA [20]. Due to limited effects of conventional disease-modifying antirheumatic drugs (DMARDs) in axial spondyloarthritis (SpA), including AS and PsA, TNF inhibition is used in SpA patients with axial disease who do not sufficiently respond to nonsteroidal anti-inflammatory drugs (NSAIDs) [21, 22]. Throughout the study period, patients using glucocorticoids were kept on a steady dose (10 mg or less per day).

### Clinical and laboratory tests

Data collection included demographic data, medical history, physical findings, lifestyle information and medication.

EF was examined, and blood samples were drawn after fasting for 8 h (including nonallowance of smoking), and hospital routine blood tests were consecutively performed.

EF was evaluated using a reactive hyperemia peripheral arterial tonometry (RH-PAT) examination which evaluates the overall health of the endothelium by measurment of finger arterial pulsatile volume changes as described previously [23]. The Reactive Hyperemic Index (RHI) was calculated as the ratio between the magnitude of the average postobstructive pulse wave amplitude (PWA) and the average of baseline PWA (preocclusion). ED was defined as RHI ≤ 1.67 as recommended by the manufacturer and in accordance with findings from a population at risk for ischemic heart disease [23]. RHI results for a subgroup of our RA sample have been published previously [24].

We evaluated improvement in RHI only in patients with ED, as a significant improvement in RHI could not be expected in patients with normal EF.

### Statistics

For comparisons of continuous independent variables between and within the examined groups, nonparametric tests (Mann-Whitney *U* test and Wilcoxon sign test) were applied, since the continuous variables of interest were not normally distributed (according to normality plots). For comparison of categorical data between the study groups, the Chi-square test was used. Linear regression analyses were used to assess associations between RHI change modeled as the dependent variable (baseline and 6 months) and selected disease activity markers. Age, gender, rheumatic diagnosis, and variables that showed a significant association with the dependent variable in simple regression analyses were included in multiple linear regression models.

*P* values ≤ 0.05 were considered statistically significant, and all statistical tests were two-sided. Our analyses were considered exploratory so no correction for multiple testing was performed.

All analyses were performed using IBM SPSS statistics, version 23.

## Results

### Baseline patient characteristics

Baseline clinical and cardiovascular characteristics of all patients who completed the 6 months of follow-up are described in Tables 1 and 2.

**Table 1:** Baseline clinical characteristics for all patients

	RA (*n* = 64)	PsA (*n* = 29)	AS (*n* = 20)	MTX (*n* = 49)	anti-TNF ± MTX (*n* = 64)
Age (years)	**57 (28–79)**	**50 (23–78)** ^**¥**^	**49 (30–72)** ^**ф**^	56 (28–79)	55 (23–75)
Women, *n* (%)	**47 (73)**	**12 (41)** ^**¥**^	**4 (20)** ^**ɸ€**^	30 (61)	33 (52)
Rheumatic disease duration (years)	2 (0–30)	3 (0–37)	3 (0–40)	**0.10 (0–25)**	3.7 (0–40)*
Disease activity					
CRP (mg/L)	8 (1–78)	5 (1–99)	10 (1–157)	8 (1–99)	6.5 (1–157)
ESR (mm/h)	**18.5 (1–81)**	**7 (2–48)** ^**¥**^	**9.5 (2–87)** ^**ɸ**^	14 (1–81)	13 (2–87)
Anti-CCP, *n* (%)	39 (61)	–	–	17 (35)	22(34)
Rheumatoid factor IgA, *n* (%)	32 (50)	–	–	15 (31)	17 (27)
Rheumatoid factor IgM, *n* (%)	45 (70)	–	–	22(45)	23(36)
BASDAI	–	4.73 (0.3–9.5)	5.1 (0.9–9.6)	5.5 (0.3–9.3)	5.1 (0.9–9.7)
BASFI	–	3.2(0–9)	4.1 (1.1–7.6)	3.0 (0–9)	3.8 (0.4–8.6)
BASMI	–	–	3 (0–10)	–	3 (0–10)
DAS28	4.98 (2.6–7.3)	–	–	5.2 (3.1–7.3)	5.1(2.6–7.1)
PtGA	52 (5–98)	44 (2–96)	56 (6–96)	52 (2–96)	49 (6–98)
PGA	**38 (7–73)**	**21 (0–57)** ^**¥**^	**26 (3–60)** ^**ф**^	**38 (11–73)**	27(0–73)*
MHAQ	0.65 (0–1.45)	0.40 (0.05–1.55)	0.43 (0–1.40)	0.45 (1–1.55)	0.50 (0–1.40)
Treatment, n (%)					
Anti-TNF monotherapy	**0 (0)**	**4 (14)** ^**¥**^	**20 (100)** ^**ɸ€**^	**0**	24 (38)*
MTX monotherapy	**34 (53)**	**15 (52)**	**0 (0)** ^**ɸ€**^	**49 (100)**	0 (0)*
Anti-TNF ± MTX	**30 (47)**	**10 (34)**	**0 (0)** ^**ɸ€**^	**0 (0)**	40 (62)*
Beta-blockers	5 (8)	1 (3)	4 (20)	4 (8)	6 (9)
Calcium antagonists	5 (8)	2 (7)	2 (10)	2 (4)	7 (11)
ACE inhibitors	6 (9)	1 (3)	2 (10)	4 (8)	5 (8)
NSAIDs	**47 (73)**	**14 (48)** ^**¥**^	**14 (70)**	35 (71)	40 (62)
Coxibs	0 (0)	0 (0)	1 (5)	0 (0)	1 (2)
Statins	**12 (19)**	**1 (3)** ^**¥**^	**7 (35)** ^**€**^	7 (14)	13 (20)
Acetyl salicylic acid	6 (9)	2 (7)	3 (15)	6 (12)	5 (8)
Warfarin	0 (0)	0 (0)	**1 (5)** ^**€**^	0 (0)	1 (2)
Glucocorticoids	**17 (27)**	**3 (10)** ^**¥**^	**2 (10)** ^**ɸ**^	**8 (16)**	15 (23)*

Unless indicated otherwise, values are given as median (range)

Statistically significant differences are shown in bold typeface

*P < 0.05, versus MTX

^¥^ *P* < 0.05, versus RA

^€^ *P* < 0.05, versus PsA

^Ф^ *P* < 0.05, versus RA

*ACE* angiotensin converting enzyme, *anti-TNF* antitumor necrosis factor, *AS* ankylosing spondylitis, *BASDAI* Bath Ankylosing Spondylitis Disease Activity Index, *BASFI* Bath Ankylosing Spondylitis Functional Index, *BASMI* Bath Ankylosing Spondylitis Metrology Index, *CRP* C-reactive protein, *DAS28* Disease activity score for 28 joints, *ED* endothelial dysfunction, *ESR* erythrocyte sedimentation rate, *Ig* immunoglobulin, *MHAQ* Medical Health Assessment Questionnaire, *MTX* methotrexate, *NSAID* nonsteroidal anti-inflammatory drug, *NSJ* number of swollen joints, *PGA* Physicians' Global Assessment Score of Disease Activity, *PsA* psoriatic arthritis, *PtGA* Patients' Global Assessment Score of Disease Activity, *RA* rheumatoid arthritis, *RHI* Reactive Hyperemic Index, *WBC* white blood cell

**Table 2:** Baseline cardiovascular characteristics for all patients

	RA (*n* = 64)	PsA (*n* = 29)	AS (*n* = 20)	MTX (*n* = 49)	anti-TNF ± MTX (*n* = 6)
Cardiovascular risk factors					
Hypertension	17 (27)	7 (24)	6 (30)	9 (18)	21 (33)
BMI (kg/m^2^), median (range)	26 (19–41)	26 (19–39)	28 (22–36)	26 (20–39)	27 (20 – 41)
Hyperlipidemia	11 (17)	3 (10)	3 (15)	9 (18)	8 (12)
Current smokers	20 (31)	6 (21)	10 (50)	15 (31)	21 (33)
Family history of CVD or death	33 (52)	13 (45)	10 (50)	24(50)	32 (50)
Diabetes	3 (5)	0 (0)	1 (5)	0 (0)	4 (6)
Medical history					
Previous myocardial infarction	5 (8)	0 (0)	2 (10)	2 (4)	5 (8)
Angina pectoris	2 (3)	1 (3)	2 (10)	2 (4)	3 (5)
Endothelial dysfunction					
RHI, median (range)	1.89 (1.24–2.94)	**2.06 (1.45–2.94)**	**1.81 (1.37–2.72)** ^**€**^	1.93 (1.24–2.76)	1.82 (1.37–2.94)
ED	22 (34)	9 (31)	9 (45)	18 (37)	22 (34)
RHI, median (range) for patients with ED	1.47 (1.24–1.65)	1.56 (1.45–1.64)	1.52 (1.37–1.64)	1.49 (1.24–1.63)	1.52 (1.37–1.65)

Unless indicated otherwise, values are given as number (percentage)

Statistically significant differences are shown in bold typeface

^€^ *P* < 0.05, versus PsA

*anti-TNF* antitumor necrosis factor, *AS* ankylosing spondylitis, *BMI* body mass index, *CVD* cardiovascular disease, *ED* endothelial dysfunction, *MTX* methotrexate, *PsA* psoriatic arthritis, *RA* rheumatoid arthritis, *RHI* Reactive Hyperemic Index

The anti-TNF ± MTX and MTX groups had similar characteristics except for a significantly shorter rheumatic disease duration and higher Physicians' Global Assessment (PGA) score in the MTX group (*p* = 0.043 and *p* = 0.002, respectively). The proportion of patients with ED was similar in both treatment groups.

Although patients with AS were the youngest (statistically significantly younger than the RA group), they had higher frequency of ED, angina pectoris, myocardial infarction, and use of some cardiovascular drugs (beta blockers, statins and warfarin) compared to the RA and PsA groups (these differences did not reach the level of statistical significance).

**Fig. 1:**
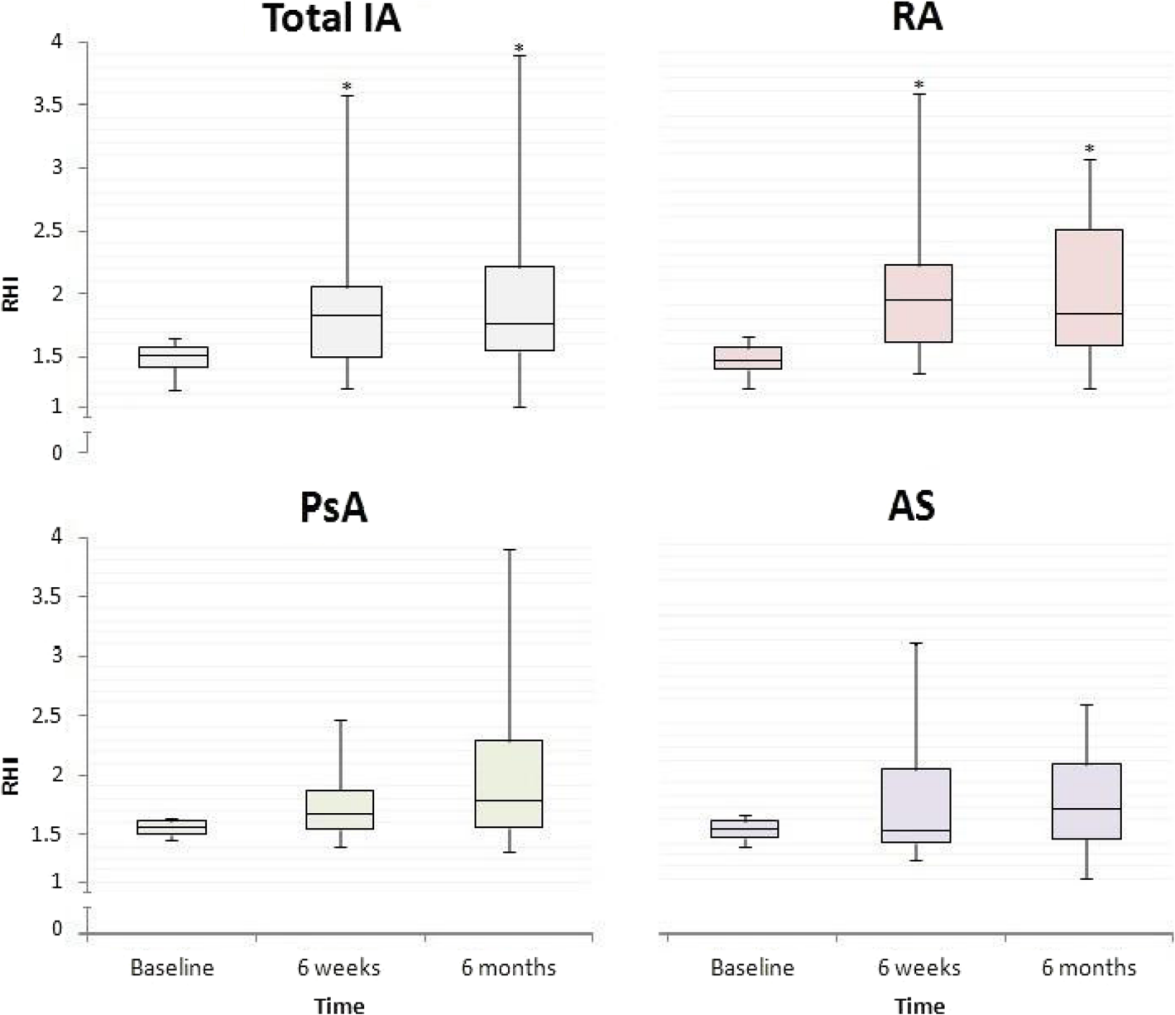
RHI values in RA, PsA, and AS patients with ED at all visits. *P < 0.05, versus baseline. The lines inside of the boxes show the median; the whiskers of the boxes show upper and lower values. *AS* ankylosing spondylitis, *IA* inflammatory arthritis, *PsA* psoriatic arthritis, *RA* rheumatoid arthritis, *RHI* Reactive Hyperemic Index

The AS group had the lowest median RHI value, which was significantly different from the PsA group (*p* = 0.040; Fig. 1). The proportion of women was highest in the RA group and lowest in the AS group (Table 1).

When evaluating only patients with ED, there were no statistically significant differences in RHI baseline values between any of the three diagnostic groups.

### RHI improvement in patients with ED

In the total IA group with ED (*n* = 40), RHI significantly improved from baseline to 6 weeks (RHI_6weeks_ = 1.86, *p* < 0.001), and from baseline to 6 months (RHI_6months_ = 1.80, *p* < 0.001) (Fig. 1). RHI baseline levels are described in Table 2.

The RHI improvement was most pronounced at 6 weeks. At 6 months, the RHI median level slightly, but statistically nonsignificantly, decreased again (Fig. 1).

In analyses of all three diagnostic groups with ED, only RA patients showed statistically significant RHI improvement from baseline to 6 weeks (RHI at 6 weeks = 1.96, *p* < 0.001) and baseline to 6 months (RHI at 6 months = 1.86, *p* = 0.001; Fig. 1). The PsA group showed RHI improvement at both visits (RHI at 6 weeks = 1.67 and RHI at 6 months = 1.80). In the AS group the RHI levels slightly decreased from baseline to 6 weeks (RHI at 6 weeks = 1.50). However, after 6 months of treatment, the RHI levels increased again (RHI at 6 months = 1.68). None of the RHI changes in the PsA and AS groups reached statistical significance.

### Effect of MTX and anti-TNF ± MTX on RHI in patients with ED

**Fig. 2:**
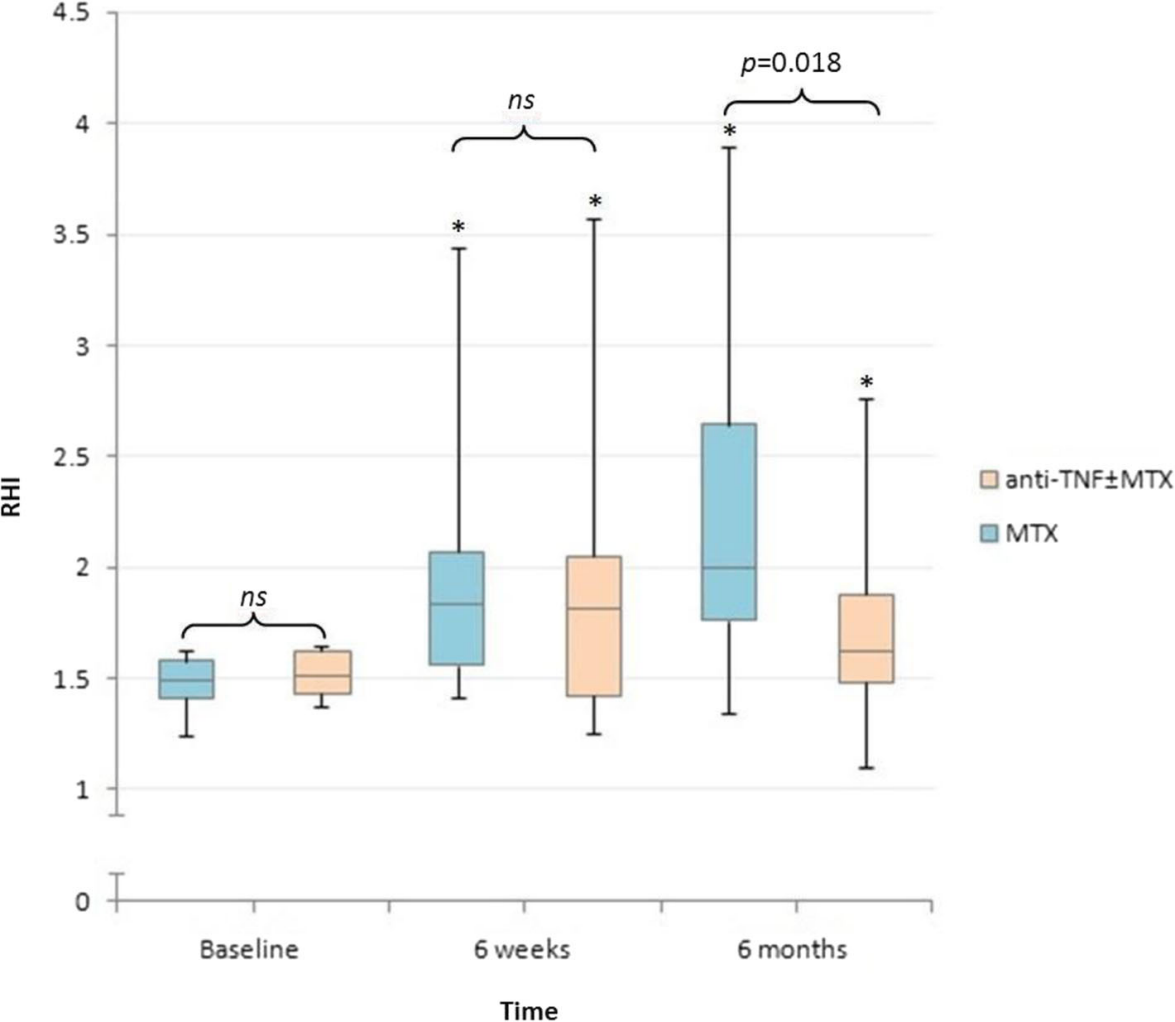
RHI values for patients with ED between and within the MTX and anti-TNF ± MTX groups. *P < 0.05 compared to baseline value. *anti-TNF* anti-tumor necrosis factor, *MTX* methotrexate, *ns* not statistically significant, *RHI* Reactive Hyperemic Index

In both treatment groups, RHI significantly improved at both follow-up visits compared to baseline (MTX: baseline to 6 weeks *p* = 0.002, baseline to 6 months *p* = 0.001; anti-TNF ± MTX: baseline to 6 weeks *p* = 0.004, baseline to 6 months *p* = 0.024). After 6 months of treatment, RHI values in the MTX group continued to increase compared to 6 weeks. However, in the anti-TNF ± MTX group RHI values at 6 months were lower than at 6 weeks, resulting in a statistically significant difference in RHI values between the two groups at 6 months (Fig. 2).

Within the RA and PsA groups there were no significant differences in RHI between patients treated with MTX and anti-TNF ± MTX.

### Linear regression analysis

Our data did not reveal any statistically significant associations between RHI and inflammatory markers, including C-reactive protein (CRP), white blood cell (WBC) count, erythrocyte sedimentation rate (ESR), pentraxin (PTX)3, Modified Health Assessment Questionnaire (MHAQ), PGA, or Patients' Global Assessment Score of Disease Activity (PtGA), at baseline (data not shown).

In simple regression analyses, only female gender and rheumatic disease duration were significantly related to RHI change from baseline to 6 months, while age, IA diagnosis, changes in markers of IA activity and severity (CRP, WBC count, ESR, PTX3, MHAQ, PGA, and PtGA) (Table 3), traditional CV risk factors (smoking, hypertension, diabetes, body mass index, established CVD (history of previous myocardial infarctions and presence of angina) and medications (statins, angiotensin converting enzyme inhibitors, and calcium antagonists); data not shown) were not.

**Table 3:** Predictors of RHI change after 6 months of antirheumatic treatment in patients with ED

	Unadjusted			Adjusted		
	Beta	*P*	95% CI	Beta	*P*	95% CI
Female gender	**0.492**	**0.011**	**0.118 to 0.865**	**0.621**	**0.004**	**0.220 to 1.022**
Age	–0.004	0.669	–0.024 to 0.016	–0.001	0.919	–0.021 to 0.019
Anti-TNF ± MTX	**–0.505**	**0.008**	**–0.866 to –0.143**	**–0.448**	**0.032**	**–0.855 to –0.041**
RDD	**–0.026**	**0.033**	**–0.049 to –0.002**	–0.024	0.068	–0.050 to 0.002
PsA	–0.067	0.779	–0.550 to 0.416	0.064	0.733	–0.386 to 0.514
AS	–0.267	0.242	–0.722 to 0.188	0.219	0.419	–0.327 to 0.765
CRP	–0.004	0.523	–0.017 to 0.009			
ESR	–0.002	0.771	–0.017 to 0.013			
PTX3	–0.071	0.255	–0.194 to 0.053			
PGA	0.008	0.160	–0.003 to 0.019			
PtGA	–0.001	0.824	–0.008 to 0.006			
MHAQ	–0.328	0.332	–1.006 to 0.349			
NSJ	–0.150	0.050	–0.299 to 0.000			

Comparators: female gender versus male gender, anti-TNF ± MTX versus MTX monotherapy

Statistically significant differences are shown in bold typeface

*anti-TNF* anti-tumor necrosis factor, *AS* ankylosing spondylitis, *CI* confidence interval, *CRP* C-reactive protein, *ESR* erythrocyte sedimentation rate, *MHAQ* Medical Health Assessment Questionnaire, *MTX* methotrexate, *NSJ* number of swollen joints, *PGA* Physicians' Global Assessment Score of Disease Activity, *PsA* psoriatic arthritis, *PtGA* Patients' Global Assessment Score of Disease Activity, *PTX3* pentraxin 3, *RDD* rheumatic disease duration, *RHI* Reactive Hyperemic Index

Female gender was related to a greater improvement in RHI compared to male gender, and the association remained statistically significant in several multiple regression models including models adjusted for age, rheumatic disease duration, and IA diagnosis. Rheumatic disease duration was negatively related to RHI change from baseline to 6 months and it stayed statistically significant in several multiple regression models (adjusted for age, gender, and IA diagnosis and age, gender, and treatment).

The difference in RHI change from baseline to 6 months between the MTX group and the anti-TNF ± MTX group remained statistically significant after adjustments for age, female gender, rheumatic disease duration, and IA diagnosis (Table 3).

### Corrections for baseline RHI values

In analyses adjusted for baseline RHI values, MTX was associated with a greater improvement in RHI than anti-TNF ± MTX after 6 months in patients with ED (*p* = 0.007).

The RHI change from baseline to 6 months was not related to RHI baseline values in patients with ED.

RHI mean values in patients with normal EF did not change at any of the control points of time (data not shown).

## Discussion

To our knowledge, this is the first study to compare the effect of MTX monotherapy and anti-TNF ± MTX treatment on EF in IA patients, and to compare levels of RHI between RA, PsA, and AS patients with active disease.

In IA patients with ED, antirheumatic treatment was associated with improvement in EF both at 6 weeks and 6 months of follow-up compared to baseline. However, after 6 weeks, EF continued to improve only in the MTX group.

Because MTX monotherapy was initiated only in MTX-naive patients, and the combination therapy only in patients who had previously used MTX without sufficient effect, our findings may indicate that MTX treatment in MTX-naive patients has a greater and more sustained vasculoprotective effect than anti-TNF monotherapy, or anti-TNF added to MTX treatment in MTX nonresponders. It is likely that, in MTX nonresponders, MTX also exhibited a poor response on disease activity after the addition of anti-TNF (MTX in this group was provided first of all to reduce side-effects of anti-TNF therapy). One might speculate that the poor response of MTX on inflammation is associated also with a poor effect on EF.

The exact mechanism behind the protective effect of antirheumatic treatment on ED is not known [25]. Theoretically, it might be mediated by inhibition of systemic inflammatory factors and the corresponding metabolic abnormalities. However, this explanation is not supported by our findings since the improvement in RHI was not related to systemic markers of disease activity, such as ESR and CRP. Moreover, we did not find any significant relationships between RHI levels and inflammatory markers at baseline.

Another explanation might be that the examined drugs might have a direct beneficial effect on the vessel walls, including the endothelium. It has been shown that MTX and anti-TNF treatments are associated with improvements in reverse cholesterol transport by various mechanisms [26, 27]. For example, MTX increases high-density lipoprotein (HDL) capacity to promote cholesterol efflux from cells [28]. Anti-TNF agents counteract the deleterious effects of TNF on the expression of genes involved in cholesterol efflux and reduce cell cholesterol accumulation through amelioration of serum lipoprotein functions and through reverse signaling following direct interaction with cell membrane-bound TNF [26].

Although most focus has been on the importance of impaired cell cholesterol efflux in the development of foam cells from macrophages in atherosclerotic plaques, the same mechanism may also underlie disturbances in endothelial cells, with reduction of their vasodilating and anti-inflammatory functions [29]. In fact, increased cholesterol efflux through the membrane transporters ATP-binding cassette A1 and G1 and Scavenger Receptor class B type I in endothelial cells is associated with promotion of eNOS expression and PGI2 production [30–32]. Moreover, serum HDL capacity to promote cell cholesterol efflux is directly correlated to flow mediated dilation [33]. Thus, the improved cell cholesterol efflux due to antirheumatic treatment might both protect from atheroma formation and from ED.

IA patients have been reported to have more inflammation, involving overexpression of TNF, in their vascular media and adventitia compared to non-IA patients with CVD [34, 35]. It might even be that inflammation located in deep vascular layers might affect the luminal part of the artery, including the phenotype of the endothelial cells [36]. Thus, in theory, antirheumatic treatment, such as anti-TNF, could also ameliorate EF by inhibition of vascular inflammation.

ED occurs when the endothelium is activated and is characterized by cytokine production, loss of vascular integrity, and expression of adhesion molecules [37]. Adhesion molecules such as intercellular adhesion molecule (ICAM)-1, E-selectin, and vascular cell adhesion molecule (VCAM)-1 make the endothelium surface more adhesive to leukocytes and facilitate their migration into the vessel wall (including atherosclerotic lesions) [37, 38].

In keeping with our results, both MTX and anti-TNF have been previously reported to downregulate expression of adhesion molecules on endothelial cells, i.e., circulating markers of ED [39–43]. Also, a recent review and meta-analysis article concluded that anti-TNF treatment might improve EF in RA patients [44].

Our previous article based on the same patient sample demonstrated that MTX and anti-TNF ± MTX treatment significantly reduced inflammatory activity (determined by ESR, CRP, WBC count, PGA, and PtGA) both at 6 weeks and at 6 months compared to baseline [16]. This may indicate that both treatment regimens have a longstanding effect on inflammation, but only MTX (in patients potentially responding to it) has a prolonged beneficial effect on the endothelial cells.

We cannot definitely rule out the possibility that the observed differences in the effect of the antirheumatic treatments on EF might be based on differences in patient populations or other factors. For example, it might be that patients with longer and more therapy-resistant IA (i.e., features typical for the anti-TNF ± MTX group; Table 1) had a higher CV risk and were less likely to improve their EF by antirheumatic treatment than the remaining IA patients (Table 2). Nevertheless, the differences in RHI change between baseline and 6 months in the two treatment groups were independent of rheumatic disease duration, IA diagnosis, and age. Moreover, there were no statistically significant differences in the examined traditional CV risk factors, the occurrences of clinical CVD and ED, and median RHI values at baseline between the two treatment groups (Table 2).

As different immune and other mechanisms are involved in the pathogenesis of RA, PsA, and AS, it might be that ED in these diseases is also mediated partly by different factors. Consequently, the effect of different antirheumatic drugs on ED in these particular diseases might also be different.

When comparing the three diagnostic groups, the AS group were the most likely to have ED and CV comorbidity (Table 2), in spite of being younger, having a similar disease duration as the RA and PsA groups, and being less likely to use systemic glucocorticoids than the RA group (Table 1). It is possible that the increased occurrence of certain CV risk factors, such as a high proportion of men and smokers, could partly explain the impaired EF in the AS group [45].

The improvements in RHI from baseline to 6 weeks and 6 months were apparent in all diagnostic groups, but it was statistically significant only in the total IA and in the RA group. The lack of statistically significant differences in RHI improvement in the other groups might be due to their relatively low sample size. Indeed, other studies indicate that antirheumatic treatment (anti-TNF) also improves EF in patients with PsA and AS [46, 47]. We cannot exclude the possibility that AS patients experienced less protection from antirheumatic treatment because they were treated only with anti-TNF and not MTX.

The cause of the observed decreased effect of anti-TNF on EF at 6 months is unclear. Among other factors, it might be caused by the well-known secondary nonresponse effect due to the development of antidrug antibodies [48].

Interestingly, women had statistically greater RHI improvement after 6 months of treatment than men (Table 2). Thus, our results may indicate that women have a better ability to reverse ED than men, independently of IA diagnosis, when treated with MTX or anti-TNF ± MTX. We do not know the molecular mechanism behind this phenomenon.

Rheumatic disease duration showed a stable negative association with RHI change from baseline to 6 months in several multiple regression models. It seems more difficult to achieve an EF improvement in patients with longer rheumatic disease duration, and this applies for both treatment regimens. Thus, these data support the notion that early antirheumatic treatment is important not only for prevention of joint damage, but also for protection from atherosclerosis. However, our results have to be confirmed in larger studies.

As in most studies, ours has several limitations. First, due to ethical reasons (to avoid prescribing MTX to patients in need of anti-TNF, and to avoid overtreatment in patients that might be sufficiently treated with MTX monotherapy) we conducted an observational study instead of a randomized controlled trial (RCT). Thus, we could not secure the same level of similarity between study groups at baseline as in a RCT, nor conduct double-blinded evaluation. On the other hand, observational studies have other advantages, e.g., they can more accurately reflect real life, and therefore have increasingly been called for over the last years. To compensate for baseline differences between the groups, we adjusted for several baseline characteristics in multiple regression models.

As MTX is the drug of choice in most patients with peripheral chronic arthritis, patients with these conditions who receive anti-TNF treatment are likely to have a longer and more severe IA. Nevertheless, the anti-TNF group did not differ from the MTX group when comparing several disease activity markers. In fact, the MTX group had statistically significantly higher PGA scores than the anti-TNF ± MTX group (Table 1).

Second, we were not able to evaluate differences in monotherapies with MTX and anti-TNF as most of the patients using anti-TNF also used MTX comedication.

Third, we evaluated RHI change only in patients with ED because we could not expect substantial RHI improvement in patients with normal EF. Therefore, regression to the mean might be questioned. However, in contrast to patients with low RHI, RHI mean values in patients with normal EF did not change towards the RHI mean at any of the control points of time. Taken together, these observations diminish the suspicion that the observed RHI differences in the ED group could be explained by regression to the mean only.

Finally, owing to a relatively small sample size, the apparent lack of some differences and associations may be due to type II errors and insufficient statistical power. Still, as this is to our knowledge the first study comparing the effect of MTX and anti-TNF regimens in IAs on EF, and comparing EF in RA, PsA, and AS, it brings new important insights into CVD in IAs, and indicates the need for further research.

An advantage of our study is a well-characterized study sample, and a design that makes it possible to examine the effect of two of the main antirheumatic treatment regimens on EF in three common IAs.

## Conclusions

In conclusion, treatment with MTX and anti-TNF ± MTX appears to improve EF relatively quickly in IA patients with ED. After 6 months, the EF improvement was more pronounced in the MTX users than in the anti-TNF ± MTX users. Among other factors, this might be due to a more sustained beneficial effect of MTX on the vasculature.

Because the EF improvement was independent of improvement in rheumatic disease activity, modes of action other than the anti-inflammatory effect might play a role.

Among patients with active RA, AS, and PsA, those with AS had the worst endothelial function (the difference was statistically significantly different compared to those with PsA), although they were the youngest.

## Abbreviations

AS: Ankylosing spondylitis
CRP: C-reactive protein
CV: Cardiovascular
CVD: Cardiovascular disease
DMARD: Disease-modifying antirheumatic drug
ED: Endothelial dysfunction
EF: Endothelial function
ESR: Erythrocyte sedimentation rate
HDL: High-density lipoprotein
IA: Inflammatory arthritis
ICAM: intercellular adhesion molecule
MHAQ: Modified Health Assessment Questionnaire
MTX: Methotrexate
NSAID: Nonsteroidal anti-inflammatory drug
PGA: Physicians' Global Assessment Score of Disease Activity
PsA: Psoriatic arthritis
PSARA: PSoriatic arthritis, Ankylosing spondylitis, Rheumatoid Arthritis
PtGA: Patients' Global Assessment Score of Disease Activity
PTX: Pentraxin
PWA: Pulse wave amplitude
RA: Rheumatoid arthritis
RCT: Randomized controlled trial
RHI: Reactive Hyperemic Index
RH-PAT: Reactive hyperemia peripheral arterial tonometry
SpA: Spondyloarthritis
TNF: Tumor necrosis factor
VCAM: Vascular cell adhesion molecule
WBC: White blood cell
